# Supplementary material for: Autologous Stem Cells in Achilles Tendinopathy (ASCAT): protocol for a phase IIA, single-centre, proof-of-concept study
Source: BMJ Open. 2018 May 14;8(5):e021600. doi: 10.1136/bmjopen-2018-021600 (PMC5961605; doi:10.1136/bmjopen-2018-021600)
Supplement: Author's manuscript [file bmjopen-2018-021600.draft_revisions.pdf]

BMJ Open

BMJ Open is committed to open peer review. As part of this commitment we make the peer review history of every article we publish publicly available.

When an article is published we post the peer reviewers' comments and the authors' responses online. We also post the versions of the paper that were used during peer review. These are the versions that the peer review comments apply to.

The versions of the paper that follow are the versions that were submitted during the peer review process. They are not the versions of record or the final published versions. They should not be cited or distributed as the published version of this manuscript.

BMJ Open is an open access journal and the full, final, typeset and author-corrected version of record of the manuscript is available on our site with no access controls, subscription charges or pay-per-view fees (http://bmjopen.bmj.com).

If you have any questions on BMJ Open's open peer review process please email info.bmjopen@bmj.com

BMJ Open

Autologous Stem Cells in Achilles Tendinopathy (ASCAT): protocol for a phase IIA, single centre, proof of concept study

Journal:	*BMJ Open*
Manuscript ID	bmjopen-2018-021600
Article Type:	Protocol
Date Submitted by the Author:	09-Jan-2018
Complete List of Authors:	Goldberg, Andrew; Royal National Orthopaedic Hospital, Foot and Ankle Unit; University College London, Senior Clinical Lecturer<br>Zaidi, Razi; Princess Royal University Hospital<br>Smith, Roger; The Royal Veterinary College<br>Brooking, Deirdre; Royal National Orthopaedic Hospital Stanmore<br>Kim, Louise ; Joint Research and Enterprise Office, St George's, University of London<br>Korda, Michelle; Queen Mary University of London<br>Masci, lorenzo; Pure Sports Medicine,<br>Green, Ruth; Royal National Orthopaedic Hospital Stanmore<br>O'Donnell, Paul; Royal National Orthopaedic Hospital Stanmore
Keywords:	Achilles tendinopathy, mesenchymal stem cell, ultrasound tissue characteristics, expanded

SCHOLARONE™
Manuscripts

For peer review only

Autologous Stem Cells in Achilles Tendinopathy (ASCAT): protocol for a phase IIA, single centre, proof of concept study

Sponsor: University College London (UCL)

Word Count - 2353

Authors:

Andy Goldberg[1] Andy.Goldberg@rnoh.nhs.uk
Razi Zaidi[2] razizaidi@doctors.net.uk
Roger Smith[3] rksmith@rvc.ac.uk
Deirdre Brooking[1] Deirdre.brooking@rnoh.nhs.uk
Louise Kim[5] lkim@sgul.ac.uk
Michelle Korda[6] michelle.korda@ucl.ac.uk
Lorenzo Masci[4] lorenzo.masci@puresportsmed.com
Ruth Green[1] ruth.green@rnoh.nhs.uk
Paul O'Donnell[1] Paul.ODonnell@rnoh.nhs.uk

[1] UCL Institute of Orthopaedics and Musculoskeletal Science (IOMS), Royal National Orthopaedic Hospital (RNOH), Brockley Hill, Stanmore HA7 4LP
[2] Princess Royal University Hospital, Farnborough Common, BR6 8ND
[3] Royal Veterinary College, Hawkshead Lane, Hatfield, AL97TA
[4] Institute of Sport Exercise and Health, 170 Tottenham Court Road, W1T 7HA
[5] Joint Research and Enterprise Office, St George's, University of London, Cranmer Terrace, SW17 0RE
[6] Queen Mary University of London, Mile End Rd, London E1 4NS.

Corresponding Author:
Andy Goldberg andy.goldberg@rnoh.nhs.uk

For peer review only

Abstract

Introduction

Achilles tendinopathy (AT) is a cause of pain and disability affecting both athletes and sedentary individuals. More than 85,000 patients present to primary care with AT annually. Whilst there is much preclinical work on the use of stem cells in tendon pathology, there is a scarcity of clinical data looking at the use of mesenchymal stem cells to treat tendon disease and there does not appear to be any studies of the use of autologous cultured mesenchymal stem cells (MSCs) for Achilles tendinopathy. Our hypothesis is that autologous culture expanded MSCs implanted into an area of midportion AT will lead to improved pain-free mechanical function. The current paper presents the protocol for a phase IIa clinical study.

Methods and analysis

The presented protocol is for a non-commercial, single arm, open-label, phase IIa proof of concept study. The study will recruit 10 participants and will follow them up for 6-months. Included will be patients aged 18 to 70 with chronic midportion AT who have failed at least 6 months of non-operative management. Participants will have a bone marrow aspirate collected from the posterior iliac crest under either local or general anaesthetic. MSCs will be isolated and expanded from the bone marrow. Four to six weeks after the harvest, participants will undergo implantation of the culture expanded MSCs under local anaesthetic and ultrasound guidance. The primary outcome will be safety as defined by the incidence rate of Serious Adverse Reaction (SAR). The secondary outcomes will be efficacy as measured by patient reported outcome measures and radiological outcome using ultrasound techniques.

For peer review only - http://bmjopen.bmj.com/site/about/guidelines.xhtml

Ethics and dissemination

The protocol has been approved by the National Research Ethics Service Committee (London, Harrow - reference 13/LO/1670). Trial findings will be disseminated through peer-reviewed publications and conference presentations.

Trial Registration

Registered on ClinicalTrials.gov 17th Febuary 2014, NCT02064062

Strengths and limitations of this study

- First study of its kind injecting culture expanded MSCs into midportion AT.
- Capturing both clinical scores and radiological outcomes.
- MSC implantation is ultrasound guided.
- Independent assessment using Ultrasound Tissue Characteristics (UTC).
- Proof of concept safety study therefore further research will be required.

For peer review only

Introduction

The Achilles tendon is the largest tendon in the body and it plays an important role in the biomechanics of the lower extremity. It can withstand great forces, especially during sporting exercise. More than 85,000 patients present to primary care with Achilles Tendinopathy (AT) annually (General Practitioner Research Database 2007). This is a degenerative process that leads to pain and dysfunction in middle-aged individuals and sports participants.[1] Tendinopathic tendons lose their shiny appearance and microscopy reveals discontinuous and disorganised collagen fibres. This is associated with mucoid and lipoid degeneration and an increasing number of cells within the tendon tissue, mostly with a fibroblastic or myofibroblastic appearance. Often, there is an abrupt discontinuity of both vascular and myofibroblastic proliferation immediately adjacent to the area of greatest abnormality.

Achilles tendon degeneration is evident as an increased signal on Magnetic Resonance Imaging (MRI)[2] and as hypoechogenic regions on ultrasound investigation.[3] These areas of abnormal imaging correspond with areas of altered collagen fibre structure and increased interfibrillar ground substance, which has been shown to consist of hydrophilic glycosaminoglycans.[2]

Achilles tendinopathy adversely affect quality of life by causing chronic pain and disability. The source of pain has been associated with neurovascular ingrowth and autocrine/paracrine effect of cellular products (e.g. catecholamines, acetylcholine, glutamate) and a reduction in pain is a key indicator of treatment success.[4]

Numerous management strategies exist for AT, such as rest, physiotherapy with eccentric strengthening exercises,[5] extracorporeal shockwave therapy,[6] dry needling,[6] high volume injections,[6] and platelet rich plasma injections.[7] However, apart from eccentric loading exercises none of these therapies have been shown to be more effective than placebo

For peer review only - http://bmjopen.bmj.com/site/about/guidelines.xhtml

(Magnussen et al, 2009).[6] A recent randomised controlled trial (RCT) demonstrated that the use of platelet-rich plasma had no statistically significant advantage over a saline placebo group. [7,8]

Although the majority of tendon conditions are treated non-operatively, up to 45% of patients at some stage consider surgery.[4] Tendon disorders are a major burden on the National Health Service (NHS). 51,454 operations on tendon disorders were performed in the NHS in 2015/16 (HESOnline), which is up from 48,765 operations performed in 2004/5. Given that the majority of tendon problems are treated non-operatively, this high surgical caseload represents just the tip of the iceberg of the real cost to the health service of tendon disease. There is therefore a need for safe and effective non-surgical treatments.

Laboratory based studies looking at autologous mesenchymal stem cells (MSCs) injections into damaged tendons have shown improved functional and histological outcomes.[9–12] Smith et al published an equine case-control trial comparing autologous bone marrow derived MSCs and saline injections. The treated tendons exhibited statistically significant improvements in key parameters with normalisation of biomechanical, morphological, and compositional parameters.[13] Another study followed up 141 horses post intralesional MSC injection for digital flexor tendinopathy. Two year follow up showed no adverse effects of the treatment with no aberrant tissue on histological examination. The re-injury percentage was significantly less than published for national hunt racehorses treated in other ways.[11]

Human studies using MSCs have shown promise in many conditions including heart disease,[14] musculoskeletal disorders,[15] autoimmune diseases,[16] and neurological disorders.[17]

With regards the use of MSCs in human tendons, there are only a handful of reports.

For peer review only

Gomes et al performed a cohort study in 14 patient that received Bone marrow concentrate (BMC) derived from the iliac crest with rotator cuff repair. 12 months following the procedure all patients had better clinical scores and MRI showed that tendons had healed.[18]

Hernigou et al performed a study comparative study looking at effect of BMC cells on rotator cuff healing post arthroscopic repair. Patients treated with BMC had a significantly increased healing rate and a significant reduction in re-rupture.[19] In contrast Mazzocca et al used BMCs from the humeral head with a similar model of rotator cuff repair, and showed no statistical difference in clinical outcome at a mean of 10.6 months.[20]

Pascual-Garrido et al looked at 8 patients with patella tendinopathy. BMCs from the iliac crest were injected into the patella tendon under ultrasound guidance. At a mean follow-up of 5 years there was significant improvement in most clinical scores. This improvement was seen up to year 2 after which it plateaued. Ultrasound evaluation was at 6 months showed that 8 of the 9 patient had improvement in the grading of the patella tendinopathy.[21]

Singh et al looked at 30 patients with untreated tennis elbow. BMCs from iliac crest were injected into the lateral epicondyle. There was a statistically significant improvement in the Patient-rated Tennis Elbow Evaluation score at 12 weeks.[22]

All of these studies used mononuclear cells from bone marrow concentrate. We could only identify one study looking at culture expanded MSCs in Achilles tendinopathy. Ilic et al reported the use of human placenta-derived allogenic MSCs injected under ultrasound guidance in 6 patients. They monitored for the primary outcome, which was safety. They concluded it was safe to inject human placenta-derived MSCs into human achilles tendons however made no comment on clinical outcome.[23]

For peer review only

There is clearly a lack of reliable evidence for the use of MSCs in tendon disease. Our hypothesis is that autologous culture expanded MSCs implanted into a tendon with Achilles tendinopathy will lead to improvement in pain-free mechanical function. The current paper presents the protocol for phase IIa trial to explore this.

Methods and Analysis

Study design

Following dialogue and engagement with the Medicines and Healthcare Regulatory Agency (MHRA) the trial was defined as a non-commercial, single arm, open-label, phase IIa proof of concept study. The study will be conducted at the Royal National Orthopaedic Hospital (RNOH). The study will recruit 10 participants and will follow them up for 6-months (Figure 1).

The primary outcome will be safety as defined by the incidence rate of Serious Adverse Reaction (SAR). The secondary outcomes will be efficacy as measured by patient reported outcome measures. Ultrasound techniques will assess the radiological outcome of the procedure. The end of the ASCAT trial is the last visit of the last participant.

Inclusion criteria

Patients aged 18 to 70 (both males and females) with chronic midportion AT (defined by pain and tender swelling in mid portion of AT (more than 1cm proximal to the insertion), with symptoms for longer than 6 months who have failed conservative treatment (at least a full course of physiotherapy) and for whom surgery is being considered. All participants must be able to provide written informed consent. Females of childbearing age and potential must be willing to use two forms of effective contraception from the time of consent to 6 months post-injection (Table 1).

For peer review only

Recruitment and consent

Patients who are referred to the RNOH will be identified as potentially eligible for the trial by the Chief Investigator (CI) or delegated clinician and invited to participate. The RNOH is a tertiary referral centre for AT and potential participants will be referred by letter to the Foot and Ankle department at the RNOH. The CI or delegated clinicians at the Foot and Ankle department will review referral letters. In the main, it may not be possible to identify relevant patients with chronic midportion AT from the referral letters; however Patient Information Sheets (PIS) may be sent by post or email to referred patients informing them of the trial. The CI or delegate will wait until the patient is seen in the initial outpatient consultation to explain the trial in more detail and if they are eligible invite them to participate. Participant Identification Centres (PIC) will also be used to identify potential patients for the trial and these may include primary care musculoskeletal triage services. Potential participants will be referred by the PIC to the trial site.

Screening Assessments

Pre-screening interview will include a medical history with discussion about willingness to participate in the trial. This will be followed by a clinical assessment that will include a and a routine examination of the foot and ankle, including; inspection of limb alignment and other biomechanical abnormality such as flat or high arched feet; size and location of swelling over the Achilles tendon and range of motion of the ankles, hindfeet, midfeet and forefeet. Assessment of gastrocnemius tightness will be performed with the knee fully straight and flexed 20 degrees (Silfverskiold test). Examination will aim to rule out other conditions such as an adjacent bursitis or insertional tendinopathy (tenderness over the calcaneal bony attachment). The Simmonds' test will be performed to ensure continuity of the tendon.

For peer review only - http://bmjopen.bmj.com/site/about/guidelines.xhtml

Blood tests will be performed to ensure compliance with the Human Tissue (Quality and Safety for Human Application) Regulations 2007 and local procedures. The minimum blood tests required as specified by the regulations will be Hepatits B(HBV), Hepatits C (HCV), Human immunodeficiency virus (HIV 1 and 2), syphilis and Human T-Cell Lymphotropic Viruses (HTLV). These tests will be carried out at least 1 week before (and within 30 days of the bone marrow harvest procedure) in line with the regulations. Additionally, a pregnancy test will be carried out prior to the bone marrow harvest (within 1 week) if the participant is a female of childbearing age and potential. If any of these tests are positive the participant will be counselled and deemed ineligible for the trial.

For peer review only

Baseline Assessment

Baseline assessments to be carried out on day 0, immediately prior to harvest include:

- Clinical assessment
- Baseline questionnaire on demographics and co-morbidity/concomitant medication
- Victorian Institute of Sports Assessment Achilles (VISA-A) questionnaire
- Visual analogue score (VAS)
- Manchester Oxford Foot & Ankle Questionnaire (MOXFQ)
- EuroQol 5-dimension 5-level (EQ-5D-5L) questionnaire
- Sporting Activity Score (SAS)
- Ultrasound imaging & Ultrasound Tissue Characteristics (UTC)
- Adverts Events (AE) review

Table 2 shows all the planned assessments and procedures that will be performed before MSC implantation.

For peer review only - http://bmjopen.bmj.com/site/about/guidelines.xhtml

Treatment Procedure

For eligible participants, approximately 8ml of bone marrow aspirate will be collected from the posterior iliac crest into a heparinized syringe under either local or general anaesthetic. A decision on which technique will be used will depend on discussions with the patient. MSC processing and manufacturing will be at the cell-processing centre at the Centre for Cell Gene and Tissue Therapeutics (CCGTT) based at the Royal Free Hospital in London. Approximately five weeks after bone marrow harvest, participants will undergo implantation of the culture expanded MSCs under local anaesthetic in the outpatient setting following clinical assessment and tendon imaging using conventional ultrasound and Ultrasound Tissue Characterisation (UTC) measurement.[24]

A single injection of between 4-20x10^6 MSCs suspended in 1ml of Dulbecco's Modified Eagles Medium (DMEM) will be injected under ultrasound control by a delegated, experienced and trained radiologist along the length of the tendon at the area of greatest degeneration starting just distal to the degeneration and finishing just proximal to normal structure. Participants will be observed for a minimum of 2 hours after implantation for any immediate adverse effects.

All participants will be referred for a standard course of physiotherapy. The physiotherapist will issue a discharge summary following their treatment and a copy of this will be included in the participant record. This summary includes details on participant compliance.

Subsequent assessments

Subsequent assessments following the MSC implantation include adverse event review, VAS (pain) score, MOXFQ, VISA-A questionnaire, EQ-5D, SAS questionnaire, ultrasound imaging (conventional ultrasound and UTC), and clinical assessment (Table 2). These will

For peer review only - http://bmjopen.bmj.com/site/about/guidelines.xhtml

occur at 6, 12 and 24 weeks post MSC implantation. After 2 days the trial coordinator will phone the patient to check they are well and record any adverse events on the ASCAT case report form. The trial will end on the last visit of the last precipitant.

Power and sample size

Sample size was determined to ensure a pre-determined level of accuracy for the incidence of Serious Adverse Reactions (SAR) using an exact binomial confidence interval. With 10 participants, if no SAR is observed we can rule out a true SAR rate of 31% or greater with 95% confidence, or a true SAR rate of 21% or greater with 80% confidence, or a true SAR rate of 13% or greater with 50% confidence. With 6 participants for the interim analysis, if no SAR is observed we can rule out a true SAR rate of 46% or greater with 95% confidence, or a true SAR rate of 32% or greater with 80% confidence, or a true SAR rate of 21% or greater with 50% confidence. Since the study is a proof of concept pilot study no formal power calculation was performed.

Statistical analysis

The findings from this study will be published and the data will also be included in subsequent larger trials if appropriate. Baseline data collected and summarised, this will include: participant gender, age, height, weight, BMI, duration of symptoms, sporting activity, smoking/non-smoking, medication, any other musculoskeletal problems, EQ-5D, MOXFQ, VAS (pain) score, SAS score, VISA-A score, and ultrasound findings (conventional ultrasound and UTC findings).

For peer review only

Continuous baseline data will be presented as mean and standard deviation for normally distributed variables, medians and interquartile ranges for non-normally distributed variables and frequencies and percentages for categorical baseline variables. For the primary outcome the proportion of participants experiencing a SAR at any time over the 24-week follow-up period will be calculated along with exact binomial confidence intervals to express the uncertainty in the observed incidence rate of SAR.

Since the primary outcome is the incidence of SARs missing outcomes are not anticipated. The primary analysis will be as per-protocol. All participants will receive the same treatment which cannot be withdrawn once given and we will only analyse the data from the participants who actually receive autologous culture expanded MSCs. Cancellation of implantation will be a logistical rather than surgical decision and as such is not expected to introduce bias. The proportion of participants experiencing a success at 6 months will be calculated along with exact binomial confidence intervals to express the uncertainty in the observed incidence of success at 6 months. Changes from baseline in EQ-5D, MOXFQ, VAS (pain) score, VISA-A and SAS scores will be summarised as mean and standard deviation if normally distributed, or median and interquartile range if the data does not demonstrate normality.

Ethics and dissemination

Ethics approval was granted 23/12/13 by the NRES Committee London- Harrow. (REC reference - 13/LO/1670). The findings of this study will be disseminated through peer-reviewed publications and conference presentations.

For peer review only

References

1 Maffulli N, Wong J, Almekinders LC. Types and epidemiology of tendinopathy. *Clin Sports Med* 2003;**22**:675–92.http://www.ncbi.nlm.nih.gov/pubmed/14560540 (accessed 18 Mar2017).

2 Movin~ T, Kristoffersen-Wiberg~ M, Rolf~ C, *et al.* ACTA R A D I O L O G I C A MR IMAGING IN CHRONIC ACHILLES TENDON DISORDER. *Acta radiol* 1998;**39**:126–32.http://www.tandfonline.com/doi/pdf/10.1080/02841859809172165 (accessed 18 Mar2017).

3 Maffulli N, Regine R, Angelillo M, *et al.* Ultrasound diagnosis of Achilles tendon pathology in runners. *Br J Sports Med* 1987;**21**:158–62.http://www.ncbi.nlm.nih.gov/pubmed/3325127 (accessed 18 Mar2017).

4 Maffulli N, Sharma P, Luscombe KL. Achilles tendinopathy: aetiology and management. *JRSM* 2004;**97**:472–6. doi:10.1258/jrsm.97.10.472

5 Alfredson H, Cook J. A treatment algorithm for managing Achilles tendinopathy: new treatment options. *Br J Sports Med* 2007;**41**:211–6. doi:10.1136/bjsm.2007.035543

6 Magnussen RA, Dunn WR, Thomson AB. Nonoperative Treatment of Midportion Achilles Tendinopathy: A Systematic Review. *Clin J Sport Med* 2009;**19**:54–64. doi:10.1097/JSM.0b013e31818ef090

7 de Vos RJ, Weir A, van Schie HTM, *et al.* Platelet-Rich Plasma Injection for Chronic Achilles Tendinopathy. *JAMA* 2010;**303**:144. doi:10.1001/jama.2009.1986

8 de Vos RJ, Weir A, Tol JL, *et al.* No effects of PRP on ultrasonographic tendon structure and neovascularisation in chronic midportion Achilles tendinopathy. *Br J Sports Med* 2011;**45**:387–92. doi:10.1136/bjsm.2010.076398

9 Hankemeier S, van Griensven M, Ezechieli M, *et al.* Tissue engineering of tendons and ligaments by human bone marrow stromal cells in a liquid fibrin matrix in immunodeficient rats: Results of a histologic study. *Arch Orthop Trauma Surg* 2007;**127**:815–21. doi:10.1007/s00402-007-0366-z

10 Nourissat G, Diop A, Maurel N, *et al.* Mesenchymal Stem Cell Therapy Regenerates the Native Bone-Tendon Junction after Surgical Repair in a Degenerative Rat Model. *PLoS One* 2010;**5**:e12248. doi:10.1371/journal.pone.0012248

11 GODWIN EE, YOUNG NJ, DUDHIA J, *et al.* Implantation of bone marrow-derived mesenchymal stem cells demonstrates improved outcome in horses with overstrain injury of the superficial digital flexor tendon. *Equine Vet J* 2012;**44**:25–32. doi:10.1111/j.2042-3306.2011.00363.x

12 Ouyang HW, Goh JCH, Lee EH. Use of Bone Marrow Stromal Cells for Tendon Graft-to-Bone Healing. *Am J Sports Med* 2004;**32**:321–7. doi:10.1177/0095399703258682

13 Smith RKW, Werling NJ, Dakin SG, *et al.* Beneficial Effects of Autologous Bone Marrow-Derived Mesenchymal Stem Cells in Naturally Occurring Tendinopathy. *doi.org* 2008;**466**:e75697. doi:10.1371/journal.pone.0075697

14 Choi Y-H, Kurtz A, Stamm C. Mesenchymal Stem Cells for Cardiac Cell Therapy. *Hum Gene Ther* 2011;**22**:3–17. doi:10.1089/hum.2010.211

15 Ahmad Z, Wardale J, Brooks R, *et al.* Exploring the Application of Stem Cells in Tendon Repair and Regeneration. *Arthrosc J Arthrosc Relat Surg* 2012;**28**:1018–29.

For peer review only - http://bmjopen.bmj.com/site/about/guidelines.xhtml

doi:10.1016/j.arthro.2011.12.009

16 Tyndall A. Successes and Failures of Stem Cell Transplantation in Autoimmune Diseases. *Hematology* 2011;**2011**:280–4. doi:10.1182/asheducation-2011.1.280

17 Uccelli A, Laroni A, Freedman MS. Mesenchymal stem cells for the treatment of multiple sclerosis and other neurological diseases. *Lancet Neurol* 2011;**10**:649–56. doi:10.1016/S1474-4422(11)70121-1

18 Ellera Gomes JL, da Silva RC, Silla LMR, *et al.* Conventional rotator cuff repair complemented by the aid of mononuclear autologous stem cells. *Knee Surgery, Sport Traumatol Arthrosc* 2012;**20**:373–7. doi:10.1007/s00167-011-1607-9

19 Hernigou P, Flouzat Lachaniette CH, Delambre J, *et al.* Biologic augmentation of rotator cuff repair with mesenchymal stem cells during arthroscopy improves healing and prevents further tears: a case-controlled study. *Int Orthop* 2014;**38**:1811–8. doi:10.1007/s00264-014-2391-1

20 Mazzocca AD, McCarthy MBR, Chowaniec DM, *et al.* Rapid Isolation of Human Stem Cells (Connective Tissue Progenitor Cells) From the Proximal Humerus During Arthroscopic Rotator Cuff Surgery. *Am J Sports Med* 2010;**38**:1438–47. doi:10.1177/0363546509360924

21 Pascual-Garrido C, Rolón A, Makino A. Treatment of chronic patellar tendinopathy with autologous bone marrow stem cells: a 5-year-followup. *Stem Cells Int* 2012;**2012**:953510. doi:10.1155/2012/953510

22 Singh A, Gangwar DS, Singh S. Bone marrow injection: A novel treatment for tennis elbow. *J Nat Sci Biol Med* 2014;**5**:389–91. doi:10.4103/0976-9668.136198

23 Ilic N, Atkinson K. Manufacturing and use of human placenta-derived mesenchymal stromal cells for phase I clinical trials: Establishment and evaluation of a protocol. *Vojnosanit Pregl* 2014;**71**:651–9. doi:10.2298/VSP130410050I

24 van Schie HTM, de Vos RJ, de Jonge S, *et al.* Ultrasonographic tissue characterisation of human Achilles tendons: quantification of tendon structure through a novel non-invasive approach. *Br J Sports Med* 2010;**44**:1153–9. doi:10.1136/bjsm.2009.061010

For peer review only

Authors' contributions

AJG, RZ, RS, LM, DB, MK, RG, LK and PD developed the trial protocol and follow-up schedule. LK and MK performed the literature review prior to grant submission. AJG, RZ, DB all contributed to the writing of the manuscript and finalizing prior to submission. AJG is the chief investigator for the study.

Funding

The trial is funded by the UK Stem Cell Foundation (Registered Charity No: 1110009).

Competing interests

None declared.

Acknowledgments

Louise Kim for helping with the grant applications, Michelle Korda for tireless efforts on the protocol, and Deirdre Brooking for the refinement of the protocol and initiation of the trial. Thanks also to Dr Hans van Schie, for his expert advice on the UTC system and to Mark Lowdell for the development of the stem cells culture protocols. We would also like to thank Iva, Ufedo and Vandana at RNOH research department for their innovation advice.

For peer review only

Tables

Table 1 Inclusion and Exclusion criteria for the study

Inclusion criteria
**1.** Aged ≥18 and ≤ 70 (both males and females)
**2.** Participants with chronic midportion AT (as defined by pain in region of AT and tender swelling in mid portion of the AT with symptoms for longer than 6 months who have failed conservative treatment (at least a full course of physiotherapy) and for whom surgery is being considered
**3.** Able to provide written informed consent

4. Females of childbearing age and potential must be willing to use two forms of effective contraception from the time of consent to 6 months post-injection.

Exclusion criteria

1. Previous bony surgery (e.g. reconstructive pelvic osteotomy) at or in proximity to the bone marrow harvest site
2. Pregnancy or lactation (self declaration at screening visit with follow up urine pregnancy test to be confirmed not beyond 7 days prior to MSC harvest and administration)
3. Current use of steroids, anti-TNF drugs, methotrexate, or ciprofloxacin (or use within 4 weeks of assessment for eligibility)
4. Positive for HBV, HCV, HIV 1 and 2, syphilis and HTLV
5. Previous AT surgery on the tendon to receive MSC implantation
6. Inflammatory arthritis
7. Known or suspected underlying haematological malignancy
8. Other active malignancy in the past 3 years
9. Bovine or antibiotic allergy (since these are in the culture medium)

For peer review only

Table 2 Schedule for assessments

	Screening	Bone Marrow Harvest	Baseline (immediately before implantation)	Implantation	Follow-up			
Visit number	1	2	3		4	5	6	7
Timing post-implantation		Approx. Week -5	Day 0		Day 2§	Week 6	Week 12	Week 24
Deviation Window (days)	**	+/-14	0		0 hours	+/-7	+/-7	+/-7
Eligibility Review	X		x					
Medical History	X							
Informed Consent	X							

Clinical Assessment	X		X					
HBV, HCV, HIV-1 & -2 and syphilis tests	X							
Pregnancy tests	X***	X	X¶					
Bone marrow harvest		X						
MSC implantation				X				
Routine course of Physiotherapy						X▲	X▲	X▲
Ultrasound Imaging (conventional ultrasound and UTC)			X^4			X^4	X^4	X^4
VISA-A questionnaire			X			X	X	X
VAS Score			X			X	X	X
MOXFQ			X			X	X	X
EQ-5D questionnaire			X			X	X	X
SAS Questionnaire		X						X
Concomitant medication	X	X	X	X	X	X	X	X
AE review		X	X	X	X	X	X	X

*** Self declaration from the patient

X Urine pregnancy test at Bone Marrow Harvest

** Screening procedures must take place at least 1 week before and within 30 days of the bone marrow procedure

§ Day 2 follow-up assessments are done by telephone

¶ Within 7 days of implantation

X^4 Two radiologists will independently scan the participant using US. UTC will also be measured twice on each occasion.

X▲The number of visits are dependent on participant's progress and are not obligatory at these time points. Standard NHS protocol for physiotherapy will start within one to two weeks after implantation. Dates of physiotherapy attendances will be recorded in the case report form (CRF).

For peer review only

Figures

Figure 1 Trial flow diagram

For peer review only

Figure 1

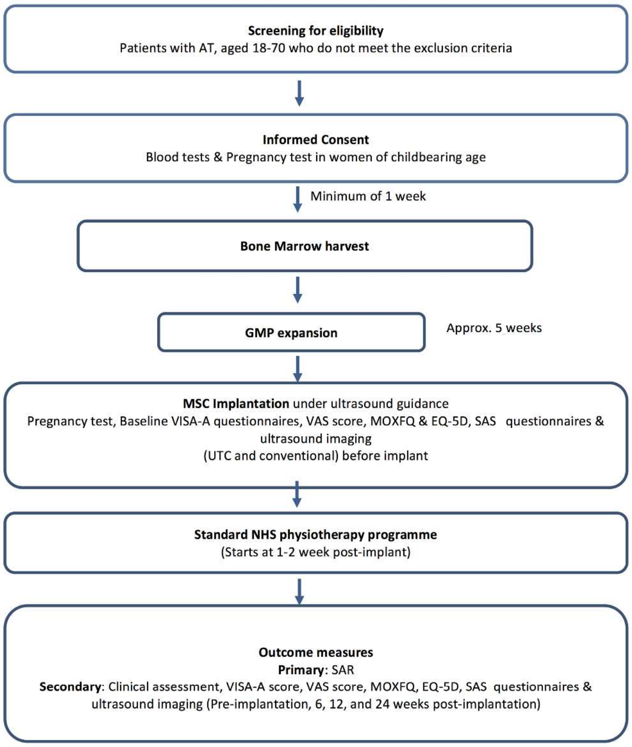

Trial flow diagram

296x420mm (300 x 300 DPI)

STROBE 2007 (v4) Statement—Checklist of items that should be included in reports of *cohort studies*

Section/Topic	Item #	Recommendation	Reported on page #
**Title and abstract**	1	(*a*) Indicate the study's design with a commonly used term in the title or the abstract	1
		(*b*) Provide in the abstract an informative and balanced summary of what was done and what was found	2
**Introduction**			
Background/rationale	2	Explain the scientific background and rationale for the investigation being reported	3-6
Objectives	3	State specific objectives, including any prespecified hypotheses	3-6
**Methods**			
Study design	4	Present key elements of study design early in the paper	7
Setting	5	Describe the setting, locations, and relevant dates, including periods of recruitment, exposure, follow-up, and data collection	17
Participants	6	(*a*) Give the eligibility criteria, and the sources and methods of selection of participants. Describe methods of follow-up	7
		(*b*) For matched studies, give matching criteria and number of exposed and unexposed	Na
Variables	7	Clearly define all outcomes, exposures, predictors, potential confounders, and effect modifiers. Give diagnostic criteria, if applicable	9-10<br>9-10
Data sources/ measurement	8*	For each variable of interest, give sources of data and details of methods of assessment (measurement). Describe comparability of assessment methods if there is more than one group	*9*
Bias	9	Describe any efforts to address potential sources of bias	7
Study size	10	Explain how the study size was arrived at	10
Quantitative variables	11	Explain how quantitative variables were handled in the analyses. If applicable, describe which groupings were chosen and why	11
Statistical methods	12	(*a*) Describe all statistical methods, including those used to control for confounding	11
		(*b*) Describe any methods used to examine subgroups and interactions	11
		(*c*) Explain how missing data were addressed	na
		(*d*) If applicable, explain how loss to follow-up was addressed	11
		(*e*) Describe any sensitivity analyses	na
**Results**			none

For peer review only

Participants	13*	(a) Report numbers of individuals at each stage of study—eg numbers potentially eligible, examined for eligibility, confirmed eligible, included in the study, completing follow-up, and analysed	7
		(b) Give reasons for non-participation at each stage	Na
		(c) Consider use of a flow diagram	18
Descriptive data	14*	(a) Give characteristics of study participants (eg demographic, clinical, social) and information on exposures and potential confounders	7
		(b) Indicate number of participants with missing data for each variable of interest	7
		(c) Summarise follow-up time (eg, average and total amount)	17
Outcome data	15*	Report numbers of outcome events or summary measures over time	9
Main results	16	(*a*) Give unadjusted estimates and, if applicable, confounder-adjusted estimates and their precision (eg, 95% confidence interval). Make clear which confounders were adjusted for and why they were included	na
		(*b*) Report category boundaries when continuous variables were categorized	na
		(*c*) If relevant, consider translating estimates of relative risk into absolute risk for a meaningful time period	na
Other analyses	17	Report other analyses done—eg analyses of subgroups and interactions, and sensitivity analyses	na
**Discussion**			
Key results	18	Summarise key results with reference to study objectives	na
**Limitations**			
Interpretation	20	Give a cautious overall interpretation of results considering objectives, limitations, multiplicity of analyses, results from similar studies, and other relevant evidence	na
Generalisability	21	Discuss the generalisability (external validity) of the study results	6
**Other information**			
Funding	22	Give the source of funding and the role of the funders for the present study and, if applicable, for the original study on which the present article is based	14

*Give information separately for cases and controls in case-control studies and, if applicable, for exposed and unexposed groups in cohort and cross-sectional studies.

Note: An Explanation and Elaboration article discusses each checklist item and gives methodological background and published examples of transparent reporting. The STROBE checklist is best used in conjunction with this article (freely available on the Web sites of PLoS Medicine at http://www.plosmedicine.org/, Annals of Internal Medicine at http://www.annals.org/, and Epidemiology at http://www.epidem.com/). Information on the STROBE Initiative is available at www.strobe-statement.org.

For peer review only

BMJ Open

Autologous Stem Cells in Achilles Tendinopathy (ASCAT): protocol for a phase IIA, single centre, proof of concept study

Journal:	*BMJ Open*
Manuscript ID	bmjopen-2018-021600.R1
Article Type:	Protocol
Date Submitted by the Author:	27-Mar-2018
Complete List of Authors:	Goldberg, Andrew; Royal National Orthopaedic Hospital NHS Trust<br>Zaidi, Razi; Princess Royal University Hospital<br>Brooking, Deirdre; Royal National Orthopaedic Hospital NHS Trust<br>Kim, Louise ; Joint Research and Enterprise Office, St George's, University of London<br>Korda, Michelle; Queen Mary University of London<br>Masci, lorenzo; Pure Sports Medicine,<br>Green, Ruth; Royal National Orthopaedic Hospital NHS Trust<br>ODonnell, Paul; Royal National Orthopaedic Hospital Stanmore<br>Smith, Roger; The Royal Veterinary College
**Primary Subject Heading**:	Sports and exercise medicine
Secondary Subject Heading:	Surgery
Keywords:	Achilles tendinopathy, mesenchymal stem cell, ultrasound tissue characteristics, expanded

SCHOLARONE™
Manuscripts

For peer review only - http://bmjopen.bmj.com/site/about/guidelines.xhtml

Autologous Stem Cells in Achilles Tendinopathy (ASCAT): protocol for a phase IIA, single centre, proof of concept study

Sponsor: University College London (UCL)

Word Count - 2708

Authors:

Andy Goldberg[1] Andy.Goldberg@nhs.net
Razi Zaidi[2] razizaidi@doctors.net.uk
Deirdre Brooking[1] Deirdre.brooking@nhs.net
Louise Kim[5] lkim@sgul.ac.uk
Michelle Korda[6] michelle.korda@ucl.ac.uk
Lorenzo Masci[4] lorenzo.masci@puresportsmed.com
Ruth Green[1] ruth.green@nhs.net
Paul O'Donnell[1] Paul.ODonnell@nhs.net
Roger Smith[3] rksmith@rvc.ac.uk

[1] UCL Institute of Orthopaedics and Musculoskeletal Science (IOMS), Royal National Orthopaedic Hospital (RNOH), Brockley Hill, Stanmore HA7 4LP
[2] Princess Royal University Hospital, Farnborough Common, BR6 8ND
[3] Royal Veterinary College, Hawkshead Lane, Hatfield, AL97TA
[4] Institute of Sport Exercise and Health, 170 Tottenham Court Road, W1T 7HA
[5] Joint Research and Enterprise Office, St George's, University of London, Cranmer Terrace, SW17 0RE
[6] Queen Mary University of London, Mile End Rd, London E1 4NS.

Corresponding Author:
Andy Goldberg andy.goldberg@nhs.net

For peer review only

Abstract

Introduction

Achilles tendinopathy (AT) is a cause of pain and disability affecting both athletes and sedentary individuals. More than 150 000 people in the United Kingdom every year suffer from AT.

Whilst there is much preclinical work on the use of stem cells in tendon pathology, there is a scarcity of clinical data looking at the use of mesenchymal stem cells to treat tendon disease and there does not appear to be any studies of the use of autologous cultured mesenchymal stem cells (MSCs) for Achilles tendinopathy. Our hypothesis is that autologous culture expanded MSCs implanted into an area of midportion AT will lead to improved pain-free mechanical function. The current paper presents the protocol for a phase IIa clinical study.

Methods and analysis

The presented protocol is for a non-commercial, single arm, open-label, phase IIa proof of concept study. The study will recruit 10 participants and will follow them up for 6-months. Included will be patients aged 18 to 70 with chronic midportion AT who have failed at least 6 months of non-operative management. Participants will have a bone marrow aspirate collected from the posterior iliac crest under either local or general anaesthetic. MSCs will be isolated and expanded from the bone marrow. Four to six weeks after the harvest, participants will undergo implantation of the culture expanded MSCs under local anaesthetic and ultrasound guidance. The primary outcome will be safety as defined by the incidence rate of Serious Adverse Reaction (SAR). The secondary outcomes will be efficacy as measured by patient reported outcome measures and radiological outcome using ultrasound techniques.

For peer review only

Ethics and dissemination

The protocol has been approved by the National Research Ethics Service Committee (London, Harrow - reference 13/LO/1670). Trial findings will be disseminated through peer-reviewed publications and conference presentations.

Trial Registration

Registered on ClinicalTrials.gov 17th Febuary 2014, NCT02064062

Strengths and limitations of this study

- A First –In-Man safety study
- First study of its kind injecting culture expanded MSCs into midportion AT.
- Capturing both clinical scores and radiological outcomes.
- Independent assessment using Ultrasound Tissue Characteristics (UTC).
- Proof of concept safety study therefore further research will be required.

For peer review only

Introduction

The Achilles tendon is the largest tendon in the body and it plays an important role in the biomechanics of the lower extremity. It can withstand great forces, especially during sporting exercise. The general population has an incidence of 2.35 per 1000 people, equivalent to more than 150 000 people in the United Kingdom every year.[1]

This is a degenerative process that leads to pain and dysfunction in middle-aged individuals and sports participants.[2] Tendinopathic tendons lose their shiny appearance and microscopy reveals discontinuous and disorganised collagen fibres. This is associated with mucoid and lipoid degeneration and an increasing number of cells within the tendon tissue, mostly with a fibroblastic or myofibroblastic appearance. Often, there is an abrupt discontinuity of both vascular and myofibroblastic proliferation immediately adjacent to the area of greatest abnormality.[3]

Achilles tendon degeneration is evident as an increased signal on Magnetic Resonance Imaging (MRI)[4] and as hypoechogenic regions on ultrasound investigation.[5] These areas of abnormal imaging correspond with areas of altered collagen fibre structure and increased interfibrillar ground substance, which has been shown to consist of hydrophilic glycosaminoglycans.[4]

Achilles tendinopathy adversely affect quality of life by causing chronic pain and disability.[6] The source of pain has been associated with neurovascular ingrowth and autocrine/paracrine effect of cellular products (e.g. catecholamines, acetylcholine, glutamate) and a reduction in pain is a key indicator of treatment success.[3]

Numerous management strategies exist for AT, such as rest, physiotherapy with eccentric strengthening exercises,[7] extracorporeal shockwave therapy,[8] dry needling,[8] high

For peer review only - http://bmjopen.bmj.com/site/about/guidelines.xhtml

volume injections,[8] and platelet rich plasma injections.[9] However, apart from eccentric loading exercises none of these therapies have been shown to be more effective than placebo (Magnussen et al, 2009).[8]

The use of PRP is controversial. One randomised controlled trial looked at PRP versus stromal vascular fraction (SVF) derived from adipose. SVF has been shown to be source of MSCs. They showed that the use of PRP and SVF was safe and that both PRP and SVF improved clinical scores with statistical significance. The use of SVF seemed to provide quicker improvement but at 180 days there was no difference between the two groups.[10] Another randomised controlled trial (RCT) demonstrated that the use of platelet-rich plasma had no statistically significant advantage over a saline placebo group. [9,11]

Although the majority of tendon conditions are treated non-operatively, up to 45% of patients at some stage consider surgery.[3] Tendon disorders are a major burden on the National Health Service (NHS). 51,454 operations on tendon disorders were performed in the NHS in 2015/16 (HESOnline), which is up from 48,765 operations performed in 2004/5. Given that the majority of tendon problems are treated non-operatively, this high surgical caseload represents just the tip of the iceberg of the real cost to the health service of tendon disease. There is therefore a need for safe and effective non-surgical treatments.

Laboratory based studies looking at autologous mesenchymal stem cells (MSCs) injections into damaged tendons have shown improved functional and histological outcomes.[12–15] Smith et al published an equine case-control trial comparing autologous bone marrow derived MSCs and saline injections. The treated tendons exhibited statistically significant improvements in key parameters with normalisation of biomechanical, morphological, and compositional parameters.[16] Another study followed up 141 horses post intralesional MSC injection for digital flexor tendinopathy. Two year follow up showed no adverse effects of the

For peer review only - http://bmjopen.bmj.com/site/about/guidelines.xhtml

treatment with no aberrant tissue on histological examination. The re-injury percentage was significantly less than published for national hunt racehorses treated in other ways.[14]

Human studies using MSCs have shown promise in many conditions including heart disease,[17] musculoskeletal disorders,[18] autoimmune diseases,[19] and neurological disorders.[20]

With regards the use of MSCs in human tendons, there are only a handful of reports. Gomes et al performed a cohort study in 14 patient that received Bone marrow concentrate (BMC) derived from the iliac crest with rotator cuff repair. 12 months following the procedure all patients had better clinical scores and MRI showed that tendons had healed.[21]

Hernigou et al performed a study comparative study looking at effect of BMC cells on rotator cuff healing post arthroscopic repair. Patients treated with BMC had a significantly increased healing rate and a significant reduction in re-rupture.[22] In contrast Mazzocca et al used BMCs from the humeral head with a similar model of rotator cuff repair, and showed no statistical difference in clinical outcome at a mean of 10.6 months.[23]

Pascual-Garrido et al looked at 8 patients with patella tendinopathy. BMCs from the iliac crest were injected into the patella tendon under ultrasound guidance. At a mean follow-up of 5 years there was significant improvement in most clinical scores. This improvement was seen up to year 2 after which it plateaued. Ultrasound evaluation was at 6 months showed that 8 of the 9 patient had improvement in the grading of the patella tendinopathy.[24]

Singh et al looked at 30 patients with untreated tennis elbow. BMCs from iliac crest were injected into the lateral epicondyle. There was a statistically significant improvement in the Patient-rated Tennis Elbow Evaluation score at 12 weeks.[25]

For peer review only - http://bmjopen.bmj.com/site/about/guidelines.xhtml

All of these studies used mononuclear cells from bone marrow concentrate. Usielli et at published a study looking at PRP and SVF. The study concluded that SVF was safe and as efficacious as PRP at 180 days, although this study did not use culture expanded MSCs .[10]. We could only identify one study looking at culture expanded MSCs in Achilles tendinopathy. Ilic et al reported the use of human placenta-derived allogenic MSCs injected under ultrasound guidance in 6 patients. They monitored for the primary outcome, which was safety. They concluded it was safe to inject human placenta-derived MSCs into human achilles tendons however made no comment on clinical outcome.[26]

There is clearly a lack of reliable evidence for the use of MSCs in tendon disease. Our hypothesis is that autologous culture expanded MSCs implanted into a tendon with Achilles tendinopathy will lead to improvement in pain-free mechanical function. The current paper presents the protocol for phase IIa trial to explore this.

Methods and Analysis

Study design

Following dialogue and engagement with the Medicines and Healthcare Regulatory Agency (MHRA) the trial was defined as a non-commercial, single arm, open-label, phase IIa proof of concept study. The study will be conducted at the Royal National Orthopaedic Hospital (RNOH). The study will recruit 10 participants and will follow them up for 6-months (Figure 1). The MHRA considered this study to be a First In Man (Phase IIa) study whose primary role was to demonstrate safety and hence all patients were to get the active treatment and no control group was used. The information from this study would then be used to design a subsequent phase III RCT involving a control.

The primary outcome will be safety as defined by the incidence rate of Serious Adverse

For peer review only - http://bmjopen.bmj.com/site/about/guidelines.xhtml

Reaction (SAR). The secondary outcomes will be efficacy as measured by patient reported outcome measures. Ultrasound techniques will assess the radiological outcome of the procedure. Computerized "Ultrasound Tissue Characterization" (UTC) is also carried out in order to add in a less subjective assessment. UTC involves a precision instrument that moves the ultrasound probe automatically across the long axis of the Achilles tendon collecting transverse images at even distances of 0.2 mm over a length of 12 cm. and enable tomographic visualization and tissue characterization and quantification of architecture and integrity of the collagenous matrix.

Ultrasound examinations undertaken in this study will performed by a musculoskeletal radiologist with experience of undertaking such an examinations. The end of the ASCAT trial is the last visit of the last participant.

Inclusion criteria

Patients aged 18 to 70 (both males and females) with chronic midportion AT (defined by pain and tender swelling in mid portion of AT (more than 1cm proximal to the insertion), with symptoms for longer than 6 months who have failed conservative treatment (at least a full course of physiotherapy) and for whom surgery is being considered. All participants must be able to provide written informed consent. Females of childbearing age and potential must be willing to use two forms of effective contraception from the time of consent to 6 months post-injection (Table 1).

Recruitment and consent

Patients who are referred to the RNOH will be identified as potentially eligible for the trial by the Chief Investigator (CI) or delegated clinician and invited to participate. The RNOH is a tertiary referral centre for AT and potential participants will be referred by letter to the Foot

For peer review only

and Ankle department at the RNOH. The CI or delegated clinicians at the Foot and Ankle department will review referral letters. In the main, it may not be possible to identify relevant patients with chronic midportion AT from the referral letters; however Patient Information Sheets (PIS) may be sent by post or email to referred patients informing them of the trial. The CI or delegate will wait until the patient is seen in the initial outpatient consultation to explain the trial in more detail and if they are eligible invite them to participate. Participant Identification Centres (PIC) will also be used to identify potential patients for the trial and these may include primary care musculoskeletal triage services. Potential participants will be referred by the PIC to the trial site.

Screening Assessments

Pre-screening interview will include a medical history with discussion about willingness to participate in the trial. This will be followed by a clinical assessment that will include a and a routine examination of the foot and ankle, including; inspection of limb alignment and other biomechanical abnormality such as flat or high arched feet; size and location of swelling over the Achilles tendon and range of motion of the ankles, hindfeet, midfeet and forefeet. Assessment of gastrocnemius tightness will be performed with the knee fully straight and flexed 20 degrees (Silfverskiold test). Examination will aim to rule out other conditions such as an adjacent bursitis or insertional tendinopathy (tenderness over the calcaneal bony attachment). The Simmonds' test will be performed to ensure continuity of the tendon.

Blood tests will be performed to ensure compliance with the Human Tissue (Quality and Safety for Human Application) Regulations 2007 and local procedures. The minimum blood tests required as specified by the regulations will be Hepatits B(HBV), Hepatits C (HCV), Human immunodeficiency virus (HIV 1 and 2), syphilis and Human T-Cell Lymphotropic

For peer review only - http://bmjopen.bmj.com/site/about/guidelines.xhtml

Viruses (HTLV). These tests will be carried out at least 1 week before (and within 30 days of the bone marrow harvest procedure) in line with the regulations. Additionally, a pregnancy test will be carried out prior to the bone marrow harvest (within 1 week) if the participant is a female of childbearing age and potential. If any of these tests are positive the participant will be counselled and deemed ineligible for the trial.

Baseline Assessment

Baseline assessments to be carried out on day 0, immediately prior to harvest include:

- Clinical assessment
- Baseline questionnaire on demographics and co-morbidity/concomitant medication
- Victorian Institute of Sports Assessment Achilles (VISA-A) questionnaire
- Visual analogue score (VAS)
- Manchester Oxford Foot & Ankle Questionnaire (MOXFQ)
- EuroQol 5-dimension 5-level (EQ-5D-5L) questionnaire
- Sporting Activity Score (SAS)
- Ultrasound imaging & Ultrasound Tissue Characteristics (UTC)
- Adverts Events (AE) review

Table 2 shows all the planned assessments and procedures that will be performed before MSC implantation.

Treatment Procedure

For eligible participants, approximately 8ml of bone marrow aspirate will be collected from the posterior iliac crest into a heparinized syringe under either local or general anaesthetic. A decision on which technique will be used will depend on discussions with the patient. MSC processing and manufacturing will be at the cell-processing centre at the Centre for Cell Gene

For peer review only - http://bmjopen.bmj.com/site/about/guidelines.xhtml

and Tissue Therapeutics (CCGTT) based at the Royal Free Hospital in London. The cells are cultured using a standardised protocol using fetal calf serum in the culture medium with robust validation systems to confirm quality and sterility of cells. Approximately five weeks after bone marrow harvest, participants will undergo implantation of the culture expanded MSCs under local anaesthetic in the outpatient setting following clinical assessment and tendon imaging using conventional ultrasound and Ultrasound Tissue Characterisation (UTC) measurement.[27]

A single injection of between 4-20x10^6 MSCs suspended in 1ml of Dulbecco's Modified Eagles Medium (DMEM) will be injected under ultrasound control by a delegated, experienced and trained radiologist along the length of the tendon at the area of greatest degeneration starting just distal to the degeneration and finishing just proximal to normal structure. Participants will be observed for a minimum of 2 hours after implantation for any immediate adverse effects.

All participants will be referred for a standard course of physiotherapy. The physiotherapist will issue a discharge summary following their treatment and a copy of this will be included in the participant record. This summary includes details on participant compliance.

Subsequent assessments

Subsequent assessments following the MSC implantation include adverse event review, VAS (pain) score, MOXFQ, VISA-A questionnaire, EQ-5D, SAS questionnaire, ultrasound imaging (conventional ultrasound and UTC), and clinical assessment (Table 2). These will occur at 6, 12 and 24 weeks post MSC implantation. After 2 days the trial coordinator will phone the patient to check they are well and record any adverse events on the ASCAT case report form. The trial will end on the last visit of the last precipitant.

For peer review only - http://bmjopen.bmj.com/site/about/guidelines.xhtml

Patient and Public Involvement

Patients and the public were not directly involved with the development of the current protocol. The primary research question is the safety of use of culture expanded MSCs. This will enable the development of a larger RCT that will involve oversight from the patient and public representatives. Patient recruitment is described above and the results will be disseminated to the participants via a news letter.

Power and sample size

Sample size was determined to ensure a pre-determined level of accuracy for the incidence of Serious Adverse Reactions (SAR) using an exact binomial confidence interval. With 10 participants, if no SAR is observed we can rule out a true SAR rate of 31% or greater with 95% confidence, or a true SAR rate of 21% or greater with 80% confidence, or a true SAR rate of 13% or greater with 50% confidence. With 6 participants for the interim analysis, if no SAR is observed we can rule out a true SAR rate of 46% or greater with 95% confidence, or a true SAR rate of 32% or greater with 80% confidence, or a true SAR rate of 21% or greater with 50% confidence. Since the study is a proof of concept pilot study no formal power calculation was performed.

Statistical analysis

The findings from this study will be published and the data will also be included in subsequent larger trials if appropriate. Baseline data collected and summarised, this will include: participant gender, age, height, weight, BMI, duration of symptoms, sporting activity, smoking/non-smoking, medication, any other musculoskeletal problems, EQ-5D, MOXFQ, VAS (pain) score, SAS score, VISA-A score, and ultrasound findings

For peer review only

(conventional ultrasound and UTC findings).

Continuous baseline data will be presented as mean and standard deviation for normally distributed variables, medians and interquartile ranges for non-normally distributed variables and frequencies and percentages for categorical baseline variables. For the primary outcome the proportion of participants experiencing a SAR at any time over the 24-week follow-up period will be calculated along with exact binomial confidence intervals to express the uncertainty in the observed incidence rate of SAR.

Since the primary outcome is the incidence of SARs missing outcomes are not anticipated. The primary analysis will be as per-protocol. All participants will receive the same treatment which cannot be withdrawn once given and we will only analyse the data from the participants who actually receive autologous culture expanded MSCs. Cancellation of implantation will be a logistical rather than surgical decision and as such is not expected to introduce bias. The proportion of participants experiencing a success at 6 months will be calculated along with exact binomial confidence intervals to express the uncertainty in the observed incidence of success at 6 months. Changes from baseline in EQ-5D, MOXFQ, VAS (pain) score, VISA-A and SAS scores will be summarised as mean and standard deviation if normally distributed, or median and interquartile range if the data does not demonstrate normality.

For peer review only

Ethics and dissemination

Ethics approval was granted 23/12/13 by the NRES Committee London- Harrow. (REC reference - 13/LO/1670). The findings of this study will be disseminated through peer-reviewed publications and conference presentations.

For peer review only

References

1 de Jonge S, van den Berg C, de Vos RJ, *et al.* Incidence of midportion Achilles tendinopathy in the general population. *Br J Sports Med* 2011;**45**:1026 LP-1028.http://bjsm.bmj.com/content/45/13/1026.abstract

2 Maffulli N, Wong J, Almekinders LC. Types and epidemiology of tendinopathy. *Clin Sports Med* 2003;**22**:675–92.http://www.ncbi.nlm.nih.gov/pubmed/14560540 (accessed 18 Mar2017).

3 Maffulli N, Sharma P, Luscombe KL. Achilles tendinopathy: aetiology and management. *JRSM* 2004;**97**:472–6. doi:10.1258/jrsm.97.10.472

4 Movin~ T, Kristoffersen-Wiberg~ M, Rolf~ C, *et al.* ACTA R A D I O L O G I C A MR IMAGING IN CHRONIC ACHILLES TENDON DISORDER. *Acta radiol* 1998;**39**:126–32.http://www.tandfonline.com/doi/pdf/10.1080/02841859809172165 (accessed 18 Mar2017).

5 Maffulli N, Regine R, Angelillo M, *et al.* Ultrasound diagnosis of Achilles tendon pathology in runners. *Br J Sports Med* 1987;**21**:158–62.http://www.ncbi.nlm.nih.gov/pubmed/3325127 (accessed 18 Mar2017).

6 Longo UG, Ronga M MN. Achilles Tendinopathy. *Sport Med Arthrosc* 2009;**17**:112–26. doi:10.1249/MSS.0b013e318227fa8c

7 Alfredson H, Cook J. A treatment algorithm for managing Achilles tendinopathy: new treatment options. *Br J Sports Med* 2007;**41**:211–6. doi:10.1136/bjsm.2007.035543

8 Magnussen RA, Dunn WR, Thomson AB. Nonoperative Treatment of Midportion Achilles Tendinopathy: A Systematic Review. *Clin J Sport Med* 2009;**19**:54–64. doi:10.1097/JSM.0b013e31818ef090

9 de Vos RJ, Weir A, van Schie HTM, *et al.* Platelet-Rich Plasma Injection for Chronic Achilles Tendinopathy. *JAMA* 2010;**303**:144. doi:10.1001/jama.2009.1986

10 Usuelli FG, Grassi M, Maccario C, *et al.* Intratendinous adipose-derived stromal vascular fraction (SVF) injection provides a safe, efficacious treatment for Achilles tendinopathy: results of a randomized controlled clinical trial at a 6-month follow-up. *Knee Surgery, Sport Traumatol Arthrosc* 2017;:1–11. doi:10.1007/s00167-017-4479-9

11 de Vos RJ, Weir A, Tol JL, *et al.* No effects of PRP on ultrasonographic tendon structure and neovascularisation in chronic midportion Achilles tendinopathy. *Br J Sports Med* 2011;**45**:387–92. doi:10.1136/bjsm.2010.076398

12 Hankemeier S, van Griensven M, Ezechieli M, *et al.* Tissue engineering of tendons and ligaments by human bone marrow stromal cells in a liquid fibrin matrix in immunodeficient rats: Results of a histologic study. *Arch Orthop Trauma Surg* 2007;**127**:815–21. doi:10.1007/s00402-007-0366-z

13 Nourissat G, Diop A, Maurel N, *et al.* Mesenchymal Stem Cell Therapy Regenerates the Native Bone-Tendon Junction after Surgical Repair in a Degenerative Rat Model. *PLoS One* 2010;**5**:e12248. doi:10.1371/journal.pone.0012248

14 GODWIN EE, YOUNG NJ, DUDHIA J, *et al.* Implantation of bone marrow-derived mesenchymal stem cells demonstrates improved outcome in horses with overstrain injury of the superficial digital flexor tendon. *Equine Vet J* 2012;**44**:25–32. doi:10.1111/j.2042-3306.2011.00363.x

15 Ouyang HW, Goh JCH, Lee EH. Use of Bone Marrow Stromal Cells for Tendon Graft-to-Bone Healing. *Am J Sports Med* 2004;**32**:321–7. doi:10.1177/0095399703258682

16 Smith RKW, Werling NJ, Dakin SG, *et al.* Beneficial Effects of Autologous Bone Marrow-Derived Mesenchymal Stem Cells in Naturally Occurring Tendinopathy. *doi.org* 2008;**466**:e75697. doi:10.1371/journal.pone.0075697

17 Choi Y-H, Kurtz A, Stamm C. Mesenchymal Stem Cells for Cardiac Cell Therapy. *Hum Gene Ther* 2011;**22**:3–17. doi:10.1089/hum.2010.211

18 Ahmad Z, Wardale J, Brooks R, *et al.* Exploring the Application of Stem Cells in Tendon Repair and Regeneration. *Arthrosc J Arthrosc Relat Surg* 2012;**28**:1018–29. doi:10.1016/j.arthro.2011.12.009

19 Tyndall A. Successes and Failures of Stem Cell Transplantation in Autoimmune Diseases. *Hematology* 2011;**2011**:280–4. doi:10.1182/asheducation-2011.1.280

20 Uccelli A, Laroni A, Freedman MS. Mesenchymal stem cells for the treatment of multiple sclerosis and other neurological diseases. *Lancet Neurol* 2011;**10**:649–56. doi:10.1016/S1474-4422(11)70121-1

21 Ellera Gomes JL, da Silva RC, Silla LMR, *et al.* Conventional rotator cuff repair complemented by the aid of mononuclear autologous stem cells. *Knee Surgery, Sport Traumatol Arthrosc* 2012;**20**:373–7. doi:10.1007/s00167-011-1607-9

22 Hernigou P, Flouzat Lachaniette CH, Delambre J, *et al.* Biologic augmentation of rotator cuff repair with mesenchymal stem cells during arthroscopy improves healing and prevents further tears: a case-controlled study. *Int Orthop* 2014;**38**:1811–8. doi:10.1007/s00264-014-2391-1

23 Mazzocca AD, McCarthy MBR, Chowaniec DM, *et al.* Rapid Isolation of Human Stem Cells (Connective Tissue Progenitor Cells) From the Proximal Humerus During Arthroscopic Rotator Cuff Surgery. *Am J Sports Med* 2010;**38**:1438–47. doi:10.1177/0363546509360924

24 Pascual-Garrido C, Rolón A, Makino A. Treatment of chronic patellar tendinopathy with autologous bone marrow stem cells: a 5-year-followup. *Stem Cells Int* 2012;**2012**:953510. doi:10.1155/2012/953510

25 Singh A, Gangwar DS, Singh S. Bone marrow injection: A novel treatment for tennis elbow. *J Nat Sci Biol Med* 2014;**5**:389–91. doi:10.4103/0976-9668.136198

26 Ilic N, Atkinson K. Manufacturing and use of human placenta-derived mesenchymal stromal cells for phase I clinical trials: Establishment and evaluation of a protocol. *Vojnosanit Pregl* 2014;**71**:651–9. doi:10.2298/VSP130410050I

27 van Schie HTM, de Vos RJ, de Jonge S, *et al.* Ultrasonographic tissue characterisation of human Achilles tendons: quantification of tendon structure through a novel non-invasive approach. *Br J Sports Med* 2010;**44**:1153–9. doi:10.1136/bjsm.2009.061010

For peer review only

Authors' contributions

AJG, RZ, RS, LM, DB, MK, RG, LK and PD developed the trial protocol and follow-up schedule. LK and MK performed the literature review prior to grant submission. AJG, RZ, DB all contributed to the writing of the manuscript and finalizing prior to submission. AJG is the chief investigator for the study.

Funding

The trial is funded by the UK Stem Cell Foundation (Registered Charity No: 1110009).

Competing interests

None declared.

Acknowledgments

Thanks go to to Dr Hans van Schie, for his expert advice on the UTC system and to Mark Lowdell for the development of the stem cells culture protocols. We would also like to thank Iva, Ufedo and Vandana at RNOH research department for their highly valuable advice and guidance.

For peer review only

Tables

Table 1 Inclusion and Exclusion criteria for the study

**Inclusion criteria**
**1.** Aged ≥18 and ≤ 70 (both males and females)
**2.** Participants with chronic midportion AT (as defined by pain in region of AT and tender swelling in mid portion of the AT with symptoms for longer than 6 months who have failed conservative treatment (at least a full course of physiotherapy) and for whom surgery is being considered
**3.** Able to provide written informed consent
**4.** Females of childbearing age and potential must be willing to use two forms of effective contraception from the time of consent to 6 months post-injection.
**Exclusion criteria**
**1.** Previous bony surgery (e.g. reconstructive pelvic osteotomy) at or in proximity to the bone marrow harvest site
**2.** Pregnancy or lactation (self declaration at screening visit with follow up urine pregnancy test to be confirmed not beyond 7 days prior to MSC harvest and administration)
**3.** Current use of steroids, anti-TNF drugs, methotrexate, or ciprofloxacin (or use within 4 weeks of assessment for eligibility)
**4.** Positive for HBV, HCV, HIV 1 and 2, syphilis and HTLV
**5.** Previous AT surgery on the tendon to receive MSC implantation
**6.** Inflammatory arthritis

For peer review only - http://bmjopen.bmj.com/site/about/guidelines.xhtml

7. Known or suspected underlying haematological malignancy
8. Other active malignancy in the past 3 years
9. Bovine or antibiotic allergy (since these are in the culture medium)

For peer review only

Table 2 Schedule for assessments

	Screening	Bone Marrow Harvest	Baseline (immediately before implantation)	Implantation	Follow-up			
Visit number	1	2	3		4	5	6	7
Timing post-implantation		Approx. Week -5	Day 0		Day 2§	Week 6	Week 12	Week 24
Deviation Window (days)	**	+/-14	0		0 hours	+/-7	+/-7	+/-7
Eligibility Review	X		x					
Medical History	X							
Informed Consent	X							
Clinical Assessment	X		X					
HBV, HCV, HIV-1 & -2 and syphilis tests	X							
Pregnancy tests	X***	X	X¶					
Bone marrow harvest		X						
MSC implantation				X				
Routine course of Physiotherapy						X▲	X▲	X▲
Ultrasound Imaging (conventional ultrasound and UTC)			X4			X4	X4	X 4
VISA-A questionnaire			X			X	X	X
VAS Score			X			X	X	X
MOXFQ			X			X	X	X

For peer review only - http://bmjopen.bmj.com/site/about/guidelines.xhtml

EQ-5D questionnaire			X			X	X	X
SAS Questionnaire		X						X
Concomitant medication	X	X	X	X	X	X	X	X
AE review		X	X	X	X	X	X	X

*** Self declaration from the patient

X Urine pregnancy test at Bone Marrow Harvest

** Screening procedures must take place at least 1 week before and within 30 days of the bone marrow procedure

§ Day 2 follow-up assessments are done by telephone

¶ Within 7 days of implantation

X[4] Two radiologists will independently scan the participant using US. UTC will also be measured twice on each occasion.

X▲The number of visits are dependent on participant's progress and are not obligatory at these time points. Standard NHS protocol for physiotherapy will start within one to two weeks after implantation. Dates of physiotherapy attendances will be recorded in the case report form (CRF).

Figures

Figure 1 Trial flow diagram

For peer review only

Figure 1

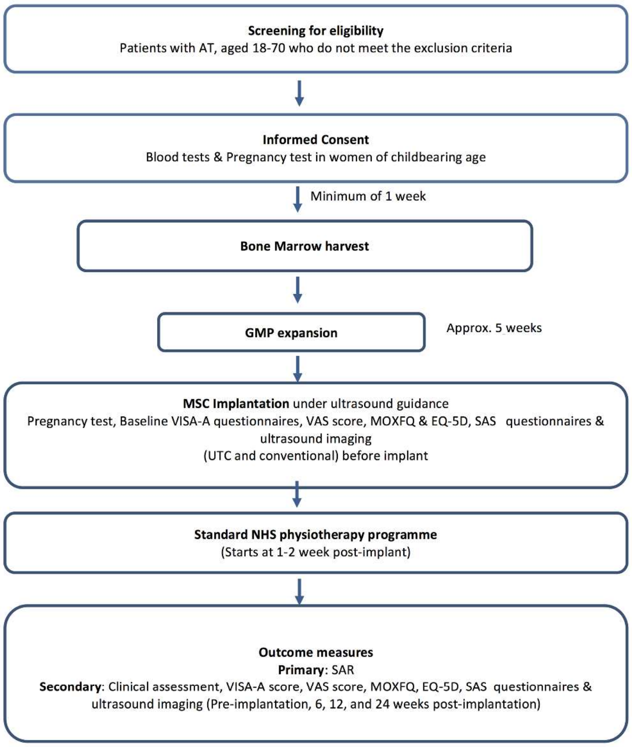

Trial flow diagram

296x420mm (300 x 300 DPI)

STROBE 2007 (v4) Statement—Checklist of items that should be included in reports of *cohort studies*

**Section/Topic**	**Item #**	**Recommendation**	**Reported on page #**
**Title and abstract**	1	(*a*) Indicate the study's design with a commonly used term in the title or the abstract	1
		(*b*) Provide in the abstract an informative and balanced summary of what was done and what was found	2
**Introduction**			
Background/rationale	2	Explain the scientific background and rationale for the investigation being reported	3-6
Objectives	3	State specific objectives, including any prespecified hypotheses	3-6
**Methods**			
Study design	4	Present key elements of study design early in the paper	7
Setting	5	Describe the setting, locations, and relevant dates, including periods of recruitment, exposure, follow-up, and data collection	17
Participants	6	(*a*) Give the eligibility criteria, and the sources and methods of selection of participants. Describe methods of follow-up	7
		(*b*) For matched studies, give matching criteria and number of exposed and unexposed	Na
Variables	7	Clearly define all outcomes, exposures, predictors, potential confounders, and effect modifiers. Give diagnostic criteria, if applicable	9-10<br>9-10
Data sources/ measurement	8*	For each variable of interest, give sources of data and details of methods of assessment (measurement). Describe comparability of assessment methods if there is more than one group	*9*
Bias	9	Describe any efforts to address potential sources of bias	7
Study size	10	Explain how the study size was arrived at	10
Quantitative variables	11	Explain how quantitative variables were handled in the analyses. If applicable, describe which groupings were chosen and why	11
Statistical methods	12	(*a*) Describe all statistical methods, including those used to control for confounding	11
		(*b*) Describe any methods used to examine subgroups and interactions	11
		(*c*) Explain how missing data were addressed	na
		(*d*) If applicable, explain how loss to follow-up was addressed	11
		(*e*) Describe any sensitivity analyses	na
**Results**			none

For peer review only

Participants	13*	(a) Report numbers of individuals at each stage of study—eg numbers potentially eligible, examined for eligibility, confirmed eligible, included in the study, completing follow-up, and analysed	7
		(b) Give reasons for non-participation at each stage	Na
		(c) Consider use of a flow diagram	18
Descriptive data	14*	(a) Give characteristics of study participants (eg demographic, clinical, social) and information on exposures and potential confounders	7
		(b) Indicate number of participants with missing data for each variable of interest	7
		(c) Summarise follow-up time (eg, average and total amount)	17
Outcome data	15*	Report numbers of outcome events or summary measures over time	9
Main results	16	(*a*) Give unadjusted estimates and, if applicable, confounder-adjusted estimates and their precision (eg, 95% confidence interval). Make clear which confounders were adjusted for and why they were included	na
		(*b*) Report category boundaries when continuous variables were categorized	na
		(*c*) If relevant, consider translating estimates of relative risk into absolute risk for a meaningful time period	na
Other analyses	17	Report other analyses done—eg analyses of subgroups and interactions, and sensitivity analyses	na
**Discussion**			
Key results	18	Summarise key results with reference to study objectives	na
**Limitations**			
Interpretation	20	Give a cautious overall interpretation of results considering objectives, limitations, multiplicity of analyses, results from similar studies, and other relevant evidence	na
Generalisability	21	Discuss the generalisability (external validity) of the study results	6
**Other information**			
Funding	22	Give the source of funding and the role of the funders for the present study and, if applicable, for the original study on which the present article is based	14

*Give information separately for cases and controls in case-control studies and, if applicable, for exposed and unexposed groups in cohort and cross-sectional studies.

Note: An Explanation and Elaboration article discusses each checklist item and gives methodological background and published examples of transparent reporting. The STROBE checklist is best used in conjunction with this article (freely available on the Web sites of PLoS Medicine at http://www.plosmedicine.org/, Annals of Internal Medicine at http://www.annals.org/, and Epidemiology at http://www.epidem.com/). Information on the STROBE Initiative is available at www.strobe-statement.org.

For peer review only
